# Anthropometric Indicators of Adiposity Related to Body Weight and Body Shape as Cardiometabolic Risk Predictors in British Young Adults: Superiority of Waist-to-Height Ratio

**DOI:** 10.1155/2018/8370304

**Published:** 2018-11-01

**Authors:** Farzad Amirabdollahian, Fahimeh Haghighatdoost

**Affiliations:** ^1^School of Health Sciences, Liverpool Hope University, Hope Park, Liverpool, UK; ^2^Department of Community Nutrition, School of Nutrition and Food Science, Isfahan University of Medical Sciences, Isfahan, Iran

## Abstract

Frequently reported poor dietary habits of young adults increase their risk of metabolic syndrome (MetS). Excess adiposity is the most established predictor of MetS, and numerous anthropometric measures have been proposed as proxy indicators of adiposity. We aimed to assess prevalence of MetS in young adult population and to make comparison between weight- and shape-oriented measures of adiposity to identify the best index in association with measured body fat and as a risk predictor for MetS. Healthy males and females aged 18–25 years from the Northwest of England were recruited using convenience sampling (*n*=550). As part of the assessment of the overall health of young adults, the biochemical variables and adiposity measures BMI, waist circumference (WC), waist-to-height ratio (WHtR), waist-to-hip ratio (WHR), new BMI, Body Adiposity Index (BAI), Clinica Universidad de Navarra-Body Adiposity Estimator (CUN-BAE), and A Body Shape Index (ABSI) were assessed. Linear regression analysis was used to investigate the association between the proxy indices of adiposity and measured percentage body fat. The odds ratio with 95% confidence interval was used to investigate the relationship between cardiometabolic (CM) risk factors and proxy measures of adiposity. The discriminatory power of these measures for diagnosis of MetS was investigated using area under the receiver operating characteristic curve. Body weight-related indicators of adiposity, particularly CUN-BAE, had stronger association with measured body fat compared with body shape-related indices. In relation with MetS, body shape-related indices, particularly elevated WC and WHtR, had stronger associations with CM risk compared with body weight-related measures. Amongst all indices, the best predictor for CM risk was WHtR, while ABSI had the weakest correlation with body fat, MetS, and CM risk. Indices directly associated with WC and specifically WHtR had greater diagnostic power in detection of CM risk in young adults.

## 1. Introduction

Emerging adulthood has been characterized with poor dietary habits [1, 2]. These poor dietary habits have been associated with the transition to independence, stress, academic and peer pressure, and taking responsibility for food choice when starting to study at university [3–5]. Several studies have reported that university students fail to meet the dietary guidelines [6–8] and gain weight in the university years [9–12], which can have adverse health consequences leading to an increased risk of obesity, type 2 diabetes, and cardiovascular diseases in later life [13, 14]. The existence of metabolic syndrome (MetS) in young adults can be a predictor of these chronic conditions in older adults [15, 16].

MetS is defined as a cluster of metabolic conditions associated with abdominal obesity including elevated blood pressure, impaired glucose tolerance, insulin resistance, elevated triglycerides, and low level of high-density lipoprotein cholesterol concentrations [17]. Similarly, the term “cardiometabolic risk” (CM) is characterized by the existence of the elements of MetS, namely, central obesity, impaired glucose metabolism, hypertension, and dyslipidaemia [18, 19]. Within the UK, several studies have investigated the prevalence and correlates of MetS in ethnic minority groups [20–23] and/or in patients with particular clinical conditions [24–28]; however, such research in emerging adulthood in the UK is scarce [29].

Amongst metabolic conditions of MetS, abdominal adiposity is of particular importance as it independently predicts the risk of other comorbidities and metabolic conditions [30]. Several anthropometric measures have been used as proxy indicators of central or whole-body adiposity.

Body mass index (BMI) developed by Adolphe Quetelet in 1832 [31] has been extensively used as a traditional proxy measure of adiposity [32]. BMI is a weight-for-height measure and by nature unable to distinguish between fat mass and muscle mass and unable to establish regional fat distribution [33]. These two substantial limitations question the discriminatory power of BMI in practice as it can potentially produce false diagnosis of adiposity, overestimate fat accumulation in tall people, and underestimate it in short people [32, 34, 35]. Furthermore, the limitation in estimating central obesity matters, as abdominal fat, is a more specific CM risk predictor compared with overall body fatness [33].

Waist circumference (WC) has been recommended with the advantage of assessing central adiposity [36, 37]; however, its application in practice has been questioned due to different cutoff points for men and women and emerging evidence showing a variation in diagnostic thresholds between ethnic groups [33, 37–40]. Similarly, the proposed ratio of waist circumference to hip circumference (WHR) as a measure of relative fat distribution requires specific gender and ethnic group cutoff points [41, 42]. Further, throughout weight loss with reduction of circumferences of both waist and hip, the ratio of waist to hip circumferences may not change substantially and therefore limits the practical utility of the measure for the CM risk management [43].

To eliminate the confounding impact of height on the association between anthropometry and CM risk [44, 45], waist-to-height ratio (WHtR) was proposed as a simple, noninvasive, and effective screening tool [46–55] benefiting from the extensive literature to support its use in relation with CM risk [56–62] and cross validation with a widely used universal cutoff point measure for identification of the abdominal obesity in different ethnic groups [63–70]. Despite this, not only has the superiority of WHtR to other anthropometric measures, as a better predictor of central adiposity and chronic diseases been questioned [71–74], but also the use of its universal yardstick for establishing central obesity in different ethnic groups has been challenged [54, 75–81].

In recent years, several body weight- and shape-associated measures of adiposity were proposed to address the limitations of the aforementioned established measures.

A correction to the equation of BMI was offered to produce a better predictor of the postoperative complications amongst colorectal cancer patients [82], but its validity and discriminatory power in relation with CM risk has yet to be tested in large samples.

Bergman et al. proposed another measure, Body Adiposity Index (BAI), calculated from hip circumference and height (Table 1) as a predictor of percentage body fat, which was validated against dual-energy X-ray absorptiometry (DXA) measurements in a large sample of Mexican adults [83]. Several studies confirmed validity and practical use of BAI [84–96]; nonetheless, an extensive body of knowledge from studies in the range of ethnic groups and patient populations questioned its validity in comparison with reference methods and/or in association with the CM risk [97–128].

The Clinica Universidad de Navarra-Body Adiposity Estimator (CUN-BAE) has been proposed to estimate percentage body fat from BMI, gender, and age [129] (Table 1); however, preliminary promising findings [130] and clinical usefulness were debated in some other studies [131–133]. A Body Shape Index (ABSI) was developed taking into consideration WC as a proxy measure of abdominal obesity but adjusting for weight and height [134]. Several studies confirmed the practical validity of ABSI [135–142]; however, others questioned its clinical use because of the limited association with measures of body fat [143], mortality [144, 145], and CM risk [146–152].

The inconsistencies, limitations, and discrepancies on reported validity of anthropometric indicators of adiposity demonstrate a gap in knowledge and a need to conduct further studies in the field. We recognise that the above anthropometric indices have been proposed as proxy indicators for total adiposity or central adiposity. Since the former invariably includes body weight and the latter typically includes waist circumference, within the current study, we categorised them as anthropometric proxy indicators related to body weight or body shape. Table 1 shows the body weight and body shape associated proxy indicators of adiposity with reference to the predictive equation model used to calculate each index.

Studies assessing the practical use of the anthropometric proxy indicators of adiposity either investigate their validity against a notional reference method or study their associations with chronic disease. The rationale for this approach is that in principle, a valid anthropometric proxy measure of adiposity must generally have a strong correlation with measured percentage body fat [153] and/or strong association with comorbidities and indeed a discriminatory power to predict their risk [154].

In the current study, to assess the predictive discriminatory power of different anthropometric measures of adiposity, we preliminarily compared them against an objective index measured, and then compared their associations with CM risk markers taking into consideration known confounding factors such as the level of physical activity. Therefore, the aim of this study was to assess prevalence of MetS in our young adult population and investigate which proxy measure of anthropometric adiposity has the strongest association (a) with measured percentage body fat and (b) with CM risk indices in healthy young adults in Northwest of England.

## 2. Materials and Methods

### 2.1. Study Design and Participants

**Figure 1:**
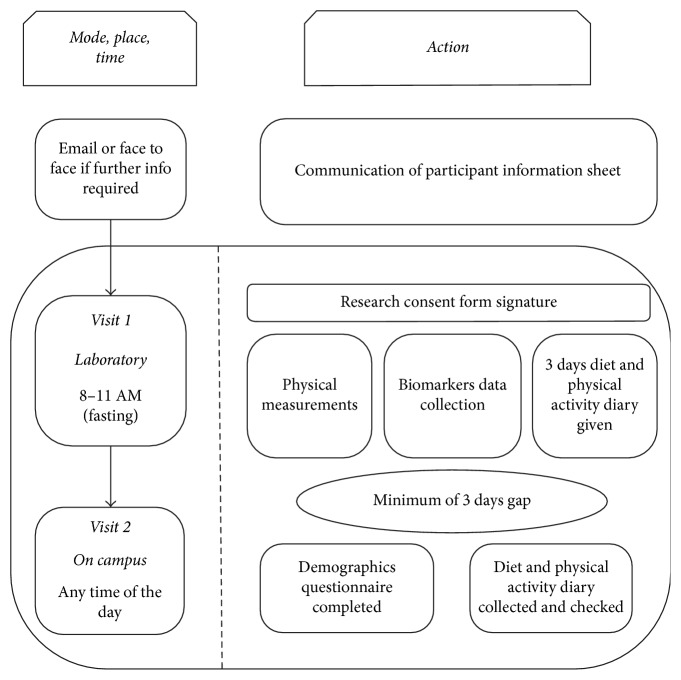
Schematic demonstrating the design of study.

Five hundred and fifty (236 male and 314 female) participants aged 18–25 years were recruited in a cross-sectional study. The study was conducted within the framework of the Collaborative Investigation on Nutritional Status of Young Adults (CINSYA) in the city of Liverpool, UK. Participants were recruited by convenience sampling from universities across the Northwest of England between 2014 and 2016 and attended two clinical visits (Figure 1). All participants gave their written informed consent for inclusion in the study, prior to participation. The study was conducted in accordance with the Declaration of Helsinki, and the protocol was approved by the Institutional Ethics Committee.

Demographic data were collected by the questionnaire using questions extracted from the validated questionnaires of the UK National Diet and Nutrition Survey (NDNS) [155].

### 2.2. Physical Measurements

Body composition, fat and fat-free mass, total body water, and the overall percentage body fat were assessed by Tanita MC-180MA, which is a multifrequency bioelectrical impedance body composition analyser (Tanita Ltd, Tokyo, Japan). For this assessment, participants were in light clothing (i.e., commonly, 0.5 kg estimated weight of the light clothing automatically deducted by the equipment), while they removed their shoes and socks before stepping on the equipment. Tanita MC-180MA also measured body mass to the nearest 0.1 kg, which was used for calculation of BMI and other anthropometric indices.

Height was determined in Frankfort plane position using a SECA 201 stadiometer (SECA GMBH & Co., Hamburg, Germany). The systolic and diastolic blood pressure (SBP and DBP, respectively) was measured in a seated position by Omron 907 professional blood pressure monitor (Omron Corporation, Kyoto, Japan) twice; in the start and toward the end of the first visit, the SBP and DBP were recorded as the mean of the two measurements. The circumferences of waist and hip were measured using nonstretchable tape measure over light clothing as advised within the literature [40].

Traditional BMI calculated as body weight divided by squared height (kg/m^2^) was classified into two categories as normal (18.5–24.9 kg/m^2^) and overweight or obese (≥25 kg/m^2^). The new BMI, which was calculated as 1.3 ∗ weight/height^2^ (kg/m^2^), was also classified into two categories using the same cutoff points: normal (18.5–24.9 kg/m^2^) and overweight or obese (≥25 kg/m^2^) [82]. ABSI [134], BAI [83], and CUN-BAE [129] were calculated based on the earlier-suggested formulae (Table 1). Since there are no population specific defined cutoff points for these three measures and also for WHR, sex-specified medians for each one was used to categorise participants into two groups (equal or more than median or lower than median). Abdominal obesity was assessed using WC and WHtR. Based on WC, participants were divided into abdominal obese, where the WC was ≥102 cm in male and ≥88 cm in female, or nonabdominally obese, where WC was <102 cm in male and <88 cm in female [156]. WHtR was calculated by dividing WC by height, which was classified as abdominal obese or nonabdominally obese using cutoff point 0.50 [50]. With regard to the cutoff points for body fat measured by the bioelectrical impedance body fat analyser, as per the manufacturer's guidelines, ≥20% of total body fat in males and ≥33% in females were considered as excessive body fat, whilst lower amounts were defined as normal values.

### 2.3. Diet and Physical Activity

A three-day integrated diet and physical activity diary were used to assess energy and nutrient intake and to estimate energy expenditure. The diet diary was extracted from the validated questionnaires of the UK's National Diet and Nutrition Survey (NDNS) [155] with minimal adjustments. To improve compliance and enhance accuracy, standardised guidelines used in NDNS, a completed example, and food portion pictures were supplied and prompts on time, place, and portion sizes were shown in the diet diary. The diaries were analysed for energy, macronutrients, and micronutrients using microdiet dietary analysis software (Microdiet v3, Downlee systems Ltd, Salford, UK). A validated 3-day physical activity diary produced by Bouchard et al. was used to assess physical activity. The analysed output of the diary produced total energy expenditure as kcal/kg/day and min/day spent in light/moderate/vigorous activity [157].

### 2.4. Biomarkers

The full procedure of capillary whole blood lipid and glucose analysis was detailed previously [158]. In brief, participants fasted overnight for least 8 hours before capillary puncture of whole blood sample was obtained. After cleaning the site with alcohol and drying it, a capillary sample of 35 *µ*l was collected using a lancet and capillary tube/plunger with heparin anticoagulant. The sample was injected into the equipment cassette, which was inserted to the analyser. The Alere LDX (Alere, San Diego, CA) was used as a point of care capillary whole blood glucose and lipid analyser to assess total cholesterol (TC), high-density lipoprotein cholesterol (HDL-C), and triglycerides (TG). These variables were then used by the analyser to calculate low-density lipoprotein cholesterol (LDL-C) using the Friedewald equation [158] and the ratio of TC/HDL. The fasting blood glucose concentration was also measured by the analyser.

### 2.5. Statistical Analysis

Data analysis was conducted using SPSS version 23 for Windows (IBM SPSS, Inc., Armonk, NY, USA). All data are expressed as mean ± standard error (SE) of mean. The required sample size (*n*=543) was estimated with 95% confidence interval and prediction of the prevalence of MetS to be around 8% (based on the midpoint of the values reported in comparable age groups [146, 159]) and with setting a margin of error and a potential for dropout. Because of the relatively large sample size of the study (*n* > 500), we did not perform the Kolmogorov–Smirnov or Shapiro–Wilk test to assess the normal distribution of the data; however, the data were scanned to remove SPSS-identified outliers based on 95% confidence interval for the mean as well as participants with improbable energy intake (i.e., reported average calorie intake <800 kcal or >4200 kcal in line with the previous literature [160]) and/or participants who did not have their anthropometric data completed. Out of 565 potential participants who contributed to study, fifteen participants were excluded as obvious outliers of normal distributions or missing key anthropometric information. Data from 550 participants (236 males and 314 females) with productive profile were used in the final analytical dataset.

Descriptive statistics were performed to establish the demographic profile of the study population. Inferential statistics were used to address the main research questions. To investigate the strengths of the association between the proxy measures of anthropometric adiposity and measured body fat, regression analysis was used.

To investigate the relationship between CM risk factors and BMI, new BMI, ABSI, CUN-BAE, BAI, WC, WHR, and WHtR, the odds ratio was calculated with 95% confidence interval from linear and logistic models in crude and adjusted models, respectively, controlling for the effect of smoking, age, and physical activity in the adjusted model. To investigate the discriminatory ability of each proxy measure of anthropometric adiposity over the possible values to detect CM risk, area under the receiver operating characteristic (ROC) curve (AUC) was quantified and tested. Further details of the statistical analysis procedure have been described previously [146].

## 3. Results

### 3.1. Participants' Characteristics

A total of 550 young adults participated in the study, and the participants' mean age and BMI were 21.2 years and 24.2 kg/m^2^, respectively. Of these participants, 57.1% were female and 96.2% were British. Most of the participants were single (80.1%), and 14.0% of them were current smoker. Means of all serum lipids, fasting blood sugar, and diastolic blood pressure were in the normal range. Mean systolic blood pressure of participants was 123.1 mmHg (Table 2).

### 3.2. Prevalence of CM Risk Factors

**Figure 2:**
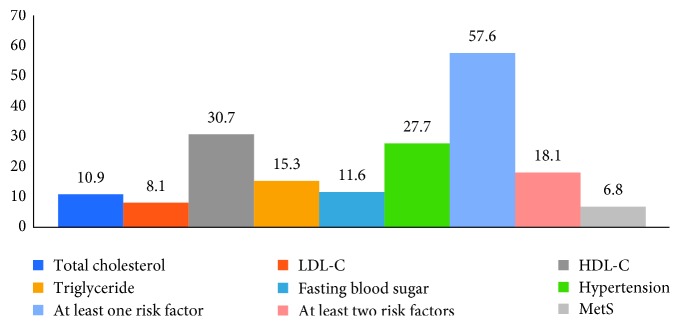
Prevalence of cardiometabolic risk in the study population.

The prevalence of CM risk factors is shown in Figure 2. Overall, 6.8% of participants were affected by MetS, 57.6% of them had at least one risk factor, and 18.1% at least two risk factors for cardiometabolic diseases. The most prevalent CM risk factor among biochemical markers was low-serum HDL-C levels (30.7%), whilst the lowest prevalent one was elevated LDL-C levels (8.1%) (Figure 2).

The prevalence of risk factors related to the biochemical test is based on total cholesterol ≥200 mg/dL, LDL ≥130 mg/dL, HDL-C <40 mg/dL in male and <50 mg/dL in female, triglyceride ≥150 mg/dL, fasting blood sugar ≥100 mg/dL, hypertension SBP ≥130.0, and/or DBP ≥85.0 mmHg. Metabolic syndrome was defined as the presence of three or more of the following components: (1) abdominal adiposity (elevated waist circumference); (2) low-serum HDL-C (<50 mg/dL); (3) high-serum triacylglycerol levels (≥150 mg/dL); (4) elevated blood pressure (≥130/85 mmHg); (5) abnormal glucose homeostasis (fasting plasma glucose level ≥110 mg/dL).

### 3.3. Association with Percentage Body Fat

The Pearson correlation coefficient from linear regression tests showed statistically significant association between all proxy anthropometric indicators of adiposity in comparison with measured percentage body fat (*P* < 0.0001). The strength of the correlation with measured body fat (%) was BMI: *r* = 0.546, new BMI: *r* = 0.589, CUN-BAE: *r* = 0.828, WC: *r* = 0.307, WHtR: *r* = 0.479, BAI: *r* = 0.681, WHR: *r* = −0.012, and ABSI: *r* = −0.426.

### 3.4. Association with Cardiometabolic Risk

The correlation between different anthropometric measurements and serum lipids and fasting blood sugar are presented in Table 3. Fasting blood sugar was positively correlated to traditional BMI, WC, WHR, and new BMI. Total cholesterol was also directly correlated with body fat percent, traditional BMI, WHtR, new BMI, CUN-BAE, and BAI. Serum TG was weakly correlated to WHR. Other biochemical risk factors were not significantly correlated with proxy anthropometric measures of adiposity.

Crude and multivariable-adjusted odds ratio (OR) and 95% CI for the presence of at least one risk factor, at least two risk factors for CM diseases, and also MetS (i.e., at least three risk factors for CM diseases) are shown in Table 4. All body weight-related or body shape-related anthropometric indicators of adiposity were associated with increased risk of having MetS, at least one risk factor or at least two risk factors of CM diseases except for ABSI. We observed that higher ABSI decreased the risk of MetS by 75% in the crude model; however, controlling for various potential confounders disappeared this association. ABSI was also not related to the risk of at least one risk factor or at least two risk factors of CM diseases. Overall, the direct link between body shape-related indicators of adiposity and CM risks was stronger than body weight-related measures. Amongst the body shape-related anthropometric indicators of adiposity, abdominal adiposity, particularly elevated WC, was the best predictor of CM risks. Amongst the body weight-related anthropometric indicators of adiposity, the best predictor for MetS was CUN-BAE, whereas the best predictor for at least one risk factor or at least two risk factors of CM diseases was the new BMI.

The ROC curve analysis examining the AUCs (and 95% CIs) of anthropometric measures in the prediction of MetS and cardiometabolic risks is shown in Table 5. The lowest AUC for all three MetS, at least one risk factor, or at least two risk factors of CM risk belonged to ABSI. Consistent with results of logistic regression, the highest AUC for MetS was related to WC and WHtR. The greatest AUC for at least two risk factors of CM was related to WHtR, which was not statistically different from body fat, BMI, WC, and new BMI. Although the highest AUC for at least one risk factor of CM also belonged to WHtR, this was not statistically different from all other indices (except for ABSI).

## 4. Discussions

To the best of our knowledge, this is the first study reporting the prevalence of CM risk and MetS in young adults in Northwest of the UK and the first that compared the association between variety of proxy indicators of adiposity with measured body fat and CM risk in this population. The study addressed its question about the clinical usefulness of different anthropometric indices of adiposity and contributes to our understanding of broad picture of nutritional status of young adults in the UK:

The current study demonstrated a significant and relatively strong correlation between most of indicators of adiposity and measured body fat and is also a strong association between these indicators and CM risk. Apart from ABSI, all other indices of adiposity were associated with CM risk when tested using multivariate-adjusted OR, while showing clear advantage for simple anthropometric indices based on waist circumference. The ROC examination also confirmed the usefulness of WC and particularly the superiority of WHtR based on the greatest AUC and therefore its diagnostic power in detection of MetS.

The strength of the correlation between CUN-BAE and measured body fat, which was in line with the previous literature [129], suggests CUN-BAE to be a potentially useful proxy measure of adiposity for our population; however, this strength was not replicated to the same extent when CUN-BAE was associated with CM risk in testing through multivariate OR and in particular, when the effect of potential confounding factors were taken into consideration in our multivariate-adjusted OR analysis. Furthermore, the prediction equation formula of CUN-BAE is rather complicated, and this limits the clinical usefulness of this measure in practice.

In addition to CUN-BAE, other indicators of anthropometric adiposity also showed statistically significant association with measured body fat, with the strength of the association declining gradually from BAI, new BMI, BMI, WHtR, WC, ABSI, and WHR, respectively; broadly showing strong correlations between body weight-related indicators of adiposity when compared with measured body fat. On the other hand, as seen with CUN-BAE, the body weight-related measures of adiposity did not produce superior association with CM risk factors limiting their clinical usefulness for the current population. Nonetheless, the strong association of the body weight-related indicators of adiposity with measured body fat was not surprising because these measures are understandably expected to have a better association with whole-body adiposity (rather than abdominal adiposity), often generated or validated based on the linear regression prediction equations against measured adiposity in cross-sectional studies [86, 94, 103, 113, 115, 125–129, 131, 132, 143], and they typically require further validation to establish their association with chronic noncommunicable diseases.

Amongst all body weight and body shape indicators of adiposity investigated, ABSI produced the weakest association with CM risk and negative association with percentage body fat. The current finding proposes that ABSI has substantial limitations for using in this population as the measure had no statistical association with CM risk factors and consequently insignificant association in multivariate-adjusted OR and the smallest AUC in the ROC curves amongst all body weight and body shape-related measures of anthropometric adiposity. This finding was in contrast with some previous studies [135, 137, 138, 142], whereas confirming some other studies [146–150]. This is difficult to explain these contrasting findings particularly in view of the different endpoint outcome variables used in different studies. For instance, the study by Krakauer and Krakauer [134] proposed ABSI as a predictor of premature mortality, whereas our study investigated the discriminatory power of this measure in distinguishing CM risk. Despite this, we thought that a potential explanation for the findings may be based on the nature and relationship between the variables used in ABSI's calculation. Conceptually, while ABSI was proposed to associate body shape with health outcomes independently of the variables defining body size (i.e., height, WC, and BMI) [134, 161], we argue that in the principle, the interrelationship between these defining variables may restrict its clinical usefulness. In particular, we support the previously proposed hypotheses that ABSI's dependency to body height may confound its capacity to distinguish CM risk in study populations [47, 48, 146, 161].

The ROC analysis demonstrates that the highest AUC belongs to WHtR confirming the discriminatory power of this measure for our target population; however, WC and new BMI also showed promising AUC for detection of MetS as potential alternatives. Similarly, the largest AUC in relation with one or two CM risk factors belonged to WHtR overall confirming the previously reported superiority of WHtR compared with other proxy measures of adiposity [52].

While the association between WHtR and measured body fat was significant, the strong association with CM risk factors in logistic regression and the excellent findings from test of the ROC curve give a collective evidence for the superiority of this measure in comparison with other anthropometric indicators of adiposity investigated. This is also important to consider the ease of use, simplicity, and clarity of the public health messages based on the WHtR (i.e., keep your waist size less than half of your height) [49], which makes it an excellent tool to be used in different settings including our population. The matter becomes more important when we consider the complexity of the prediction equation formulae of some of the investigated proxy indicators of adiposity and the necessity for establishing gender and ethnicity specific cutoff points in order to interpret their findings.

The pathophysiological mechanism to explain the association between WHtR and CM risk is yet to be fully elucidated. Height can reflect early life exposures. A prospective cohort study among Chilean adults suggests that individuals who had adverse environmental exposures during childhood were more likely to have short stature and abdominal adiposity, insulin resistance, and other CM risk factors in their adulthood [162]. In addition, due to inverse associations between height and mortality and CM morbidity, inclusion of the parameter of stature beside central adiposity measures such as WC might be the reason for the superiority of WHtR over BMI and WC [163, 164]. Alternatively, height may not only affect CM risk via independent mechanisms but also it might change CM risk via affecting other mediators. Schneider et al. indicated greater CM risk in short subjects compared with tall subjects when they were grouped by WC, but not WHtR, which suggests that these differences cannot be explained by height alone [165]. Moreover, it should be taken into account that height remains relatively unchanged during adulthood, and therefore, WHtR will change only by the changes in the waist measurement, whereas indices like waist-to-hip ratio are more likely to be changed over time since body size is changing, and consequently, hip and waist circumference would increase or decrease proportionately [52].

The current study has some limitations and strengths to be considered in the interpretation of the results and in the design and conduct of the future similar investigations:

The present study examined participants recruited through nonrandomized convenient sampling who might not be a fully representative sample of young adults residing in the NW of England. This may limit the generalizability of our findings, and the results should be interpreted carefully and in view of the above limitation. It is also important to consider that within this study, we only examined a group of anthropometric indicators of adiposity, and our analysis by no means has included all possible proxy indicators and data analysis approaches. For instance, a measure such as Body Roundness Index (BRI) [166] has recently produced promising indications of clinical use; however, the investigation of BRI did not fully fit the overall conceptual framework of the current study as the prediction equation includes body weight and body shape-related parameters within the same measure. Future studies should not only investigate the use of BRI but also consider other proxy indicators such Anthropometric Risk Index (ARI) [167] and Conicity Index (CI) [168], while implementing *Z-*score to adjust for age and gender variation in larger and more diverse populations.

Although we did not stratify our analysis by gender, its confounding effect was controlled. In addition, due to differences in fat and lean mass tissue between men and women, we categorized participants based on gender-specific cutoff points for anthropometric measures, which do not have predefined threshold that minimize its confounding effect.

In the current study and in consideration of our sample size, use of a lab-based objective reference method of assessment of body composition such as magnetic resonance imaging (MRI), dual-energy X-ray absorptiometry (DXA), and/or hydrodensitometry [169] was not plausible, and hence, we used a multifrequency bioelectrical impedance body composition analyzer with an advanced functionality to measure percentage body fat, for which the validity and clinical usefulness was demonstrated in previous studies [170, 171].

The current investigation measured basic anthropometric indices, and this was an advantage compared with some previous investigations using self-reported anthropometry. The assessors of anthropometry in this study were graduate nutritionists, who were trained by a qualified registered nutritionist with experience of assessment of anthropometry. The body weight and body shape-related measures used in the analysis were computed electronically, and this restricted the potential impact of the human error.

Although we considered various potential confounding factors in our analysis, the aetiology of the MetS and occurrence of the CM risk in the population is heterogonous and yet to be fully understood. Future studies should therefore control for a wider range of potential confounders including dietary, socioeconomic, and biochemical measures to elucidate the associations between anthropometric proxy measures and CM risk with elimination of potential mediating and moderating factors.

## 5. Conclusions

Overall, most of the body weight and body shape-related indicators of anthropometric adiposity showed statistically significant association with measured body fat and with CM risk factors. Body weight-related measures such as CUN-BAE and new BMI demonstrated strong association with measured adiposity but did not show adequate discriminatory power in identifying CM risk. On the other hand, body shape-related indicators of anthropometric adiposity such as BAI, WC, and WHtR showed mediocre association with measured percentage body fat and superior discriminatory power in identifying CM risk.

Overall, the acceptable statistically significant association of the WHtR against measured body fat and the strong association with CM risk in logistic regression and the AUC when testing the ROC curve of the WHtR, together with simplicity and clarity of the public health message in communication of the use of the measure, are amongst the reasons to propose WHtR as a clinically superior measure of anthropometric adiposity for our population. We therefore recommend the use of WHtR as a simple, effective, and clear measure for monitoring the CM risk within young adults.

## Figures and Tables

**Table 1:** Anthropometric indicators of adiposity used in this study, reference, and equation for calculation.

Measure	Author (year)	Equation
*Anthropometric indicators of adiposity related to body weight*		
Body mass index (BMI)	Gysel (1974) [31]	BMI = body weight (kg)/height (m)^2^
New body mass index (New BMI)	Van Vugt et al. (2015) [82]	New BMI = 1.3 × (weight (kg)/height (m)^2^)
Clinica Universidad de Navarra-Body Adiposity Estimator (CUN-BAE)	Gomez-Ambrosi et al. (2012) [129]	BF% = −44.988 + (0.503 × age (years)) + (10.689 × sex) + (3.172 × BMI (kg/m^2^))−(0.026 × BMI^2^ (kg/m^2^)) + (0.181 × BMI (kg/m^2^) × sex) − (0.02 × BMI (kg/m^2^) × age) − (0.005 × BMI^2^ (kg/m^2^) × sex) + (0.00021 × BMI^2^ (kg/m^2^) × age), where male = 0 and female = 1

*Anthropometric indicators of adiposity related to body shape*		
Waist circumference (WC)	WHO (2008) [37]	Circumference of the waist measured in standardized position as advised by the WHO (cm)
Waist-to-hip ratio (WHR)	WHO (2008) [37]	WHR = waist circumference (cm)/hip circumference (cm)
Waist-to-height ratio (WHtR)	Ashwell (1995) [46]	WHtR = waist circumference (cm)/height (cm)
Body Adiposity Index (BAI)	Bergman et al. (2011) [83]	BAI (percentage body fat, BF%) = (hip circumference (cm)/height (m)^1.5^) − 18.
A Body Shape Index (ABSI)	Krakauer and Krakauer (2012) [134]	ABSI = waist circumference (cm)/(BMI (kg/m^2^)^0.66^ × height (m)^0.5^)

**Table 2:** Characteristics of the study population.

Index	Mean ± SE or %
Age (years)	21.19 ± 0.10
BMI (kg/m^2^)	24.18 ± 0.18
Body fat (%)	24.60 ± 0.39
WC (cm)	80.51 ± 0.50
CUN-BAE	26.03 ± 0.36
BAI	45.08 ± 0.22
ABSI	0.00030 ± 0.0000035
New BMI (kg/m^2^)	24.11 ± 0.18
WHR	0.80 ± 0.003
WHtR	0.47 ± 0.003
Total cholesterol (mg/dL)	158.70 ± 1.49
LDL-C (mg/dL)	104.83 ± 16.56
HDL-C (mg/dL)	59.30 ± 5.59
Triglyceride (mg/dL)	115.44 ± 12.07
Fasting blood sugar (mg/dL)	89.82 ± 0.50
Systolic blood pressure (mmHg)	123.14 ± 0.61
Diastolic blood pressure (mmHg)	75.59 ± 0.45
Female (%)	57.1
British (home students) (%)	96.2
Single (%)	80.1
Smoker (%)	14.0
Population at risk based on BMI^*∗*^ (%)	32.5
Population at risk based on new BMI^*∗*^ (%)	31.8
Population at risk based on WC^*∗*^ (%)	12.2
Population at risk based on WHtR^*∗*^ (%)	28.5
Population at risk based on excessive measured body fat^*∗*^ (%)	32.7

^*∗*^Percentage of the population classified at risk is calculated using accepted boundary values for the anthropometric indices: BMI/new BMI ≥ 25 kg/m^2^, abdominal obese: WC ≥ 102 cm in males and ≥88 cm in females, elevated WHtR: ≥0.5, and excess body fat: body fat ≥20% in male and ≥33% in female.

**Table 3:** Linear regression of proxy anthropometric measures of adiposity with cardiometabolic risk.^1^

	Blood sugar	Total cholesterol	LDL-C	HDL-C	TG
Body fat percent	0.027	0.173^§^	−0.004	−0.016	0.026
BMI	0.131^§^	0.103^§^	−0.006	−0.049	0.052
New BMI	0.106^§^	0.129^§^	−0.004	−0.033	0.054
CUN-BAE	0.001	0.177^§^	0.024	−0.052	0.037
WC	0.236^§^	0.069	0.025	−0.052	0.055
WHR	0.208^§^	0.032	0.013	0.022	0.087^§^
WHtR	0.195	0.126^§^	0.040	−0.031	0.061
BAI	0.028	0.160^§^	0.050	−0.036	0.005
ABSI	−0.070	−0.078	0.028	0.048	−0.040

^1^Using linear regression;^§^*P* < 0.05.

**Table 4:** Multivariate-adjusted odds ratio (and 95% confidence intervals) for cardiometabolic risk associated with proxy indicators of anthropometric adiposity.^1^

	MetS	*P* value^2^	At least two risk factors	*P* value^2^	At least one risk factor	*P* value^2^
*Crude model*						
Body fat percent	7.47 (3.44, 16.22)	<0.0001	2.25 (1.42, 3.54)	<0.0001	1.96 (1.33, 2.89)	0.001
BMI	10.50 (4.51, 24.43)	<0.0001	2.62 (1.66, 4.14)	<0.0001	2.36 (1.59, 3.51)	<0.0001
New BMI	9.23 (4.12, 20.66)	<0.0001	2.87 (1.82, 4.54)	<0.0001	2.71 (1.81, 4.07)	<0.0001
CUN-BAE	12.81 (3.88, 42.24)	<0.0001	1.82 (1.15, 2.87)	0.010	1.92 (1.34, 2.74)	<0.0001
WC	32.40 (14.62, 71.80)	<0.0001	2.85 (1.60, 5.08)	<0.0001	3.21 (1.69, 6.07)	<0.0001
WHR	16.49 (3.92, 69.31)	<0.0001	2.05 (1.28, 3.30)	0.003	1.43 (1.00, 2.04)	0.048
WHtR	26.32 (9.14, 75.78)	<0.0001	2.98 (1.87, 4.73)	<0.0001	2.92 (1.90, 4.49)	<0.0001
BAI	9.32 (3.26, 26.71)	<0.0001	1.80 (1.14, 2.85)	0.011	1.79 (1.26, 2.56)	0.001
ABSI	0.25 (0.11, 0.56)	0.001	0.71 (0.45, 1.11)	0.137	0.72 (0.50, 1.02)	0.066

*Adjusted model*						
Body fat percent	5.33 (2.36, 12.07)	<0.0001	1.90 (1.16, 3.14)	0.011	1.89 (1.24, 2.89)	0.003
BMI	7.99 (3.15, 20.32)	<0.0001	2.19 (1.27, 3.78)	0.005	2.54 (1.58, 4.07)	<0.0001
New BMI	6.60 (2.73, 15.95)	<0.0001	2.37 (1.39, 4.05)	0.002	2.96 (1.84, 4.75)	<0.0001
CUN-BAE	9.02 (2.57, 31.73)	0.001	1.37 (0.79, 2.35)	0.258	1.88 (1.22, 2.90)	0.004
WC	58.04 (18.30, 184.10)	<0.0001	2.65 (1.38, 5.09)	0.003	2.96 (1.50, 5.86)	0.002
WHR	16.26 (3.77, 70.12)	<0.0001	1.83 (1.09, 3.06)	0.021	1.45 (0.98, 2.15)	0.061
WHtR	20.88 (7.00, 62.29)	<0.0001	2.55 (1.53, 4.24)	<0.0001	3.07 (1.91, 4.91)	<0.0001
BAI	6.91 (2.37, 20.16)	<0.0001	1.62 (1.00, 2.63)	0.050	1.77 (1.22, 2.58)	0.003
ABSI	0.41 (0.17, 1.01)	0.052	0.99 (0.58, 1.67)	0.961	0.83 (0.55, 1.25)	0.374

^1^Using logistic regression; ^2^derived from a Mantel–Haenszel extension chi-square test.

**Table 5:** Area under curve analysis for cardiometabolic risk associated with body weight and body shape-related indicators of adiposity.

	MetS	At least two risk factors	At least one risk factor
Body fat percent	0.771 (0.694, 0.847)	0.605 (0.544, 0.665)	0.595 (0.545, 0.644)
BMI	0.827 (0.747, 0.906)	0.614 (0.548, 0.680)	0.615 (0.566, 0.663)
New BMI	0.826 (0.746, 0.907)	0.637 (0.572, 0.702)	0.630 (0.582, 0.678)
CUN-BAE	0.768 (0.680, 0.856)	0.596 (0.530, 0.661)	0.600 (0.551, 0.650)
WC	0.889 (0.831, 0.947)	0.640 (0.575, 0.705)	0.612 (0.563, 0.660)
WHR	0.782 (0.723, 0.841)	0.638 (0.575, 0.701)	0.585 (0.536, 0.634)
WHtR	0.892 (0.831, 0.953)	0.663 (0.600, 0.727)	0.631 (0.583, 0.680)
BAI	0.776 (0.692, 0.860)	0.581 (0.517, 0.646)	0.599 (0.549, 0.648)
ABSI	0.233 (0.140, 0.327)	0.427 (0.358, 0.495)	0.415 (0.365, 0.464)

## Data Availability

We will obviously be more than happy to submit our data to the Journal reviewers if necessary; however, the data will not be available to public as further manuscripts are expected to be submitted from the analysis of the data.
